# Infectious disease burden and surveillance challenges in Jordan and Palestine: a systematic review and meta-analysis

**DOI:** 10.3389/fdgth.2025.1713089

**Published:** 2026-01-29

**Authors:** Eman Farouq Badran, Abdullah Rayyan, Mira Al Jaberi, Muayyed Azzam, Raghad Ramadan, Yousef Khader, Raeda Alqutob, Faris G. Bakri, Radwan Qasrawi, Taimein Yacoub, Areej Sharaqa, Nadine Fraihat, Hana Trigui, Elie Sokhn, Reema Tayyem, Emmanuel Musa, Jude Dzevela Kong

**Affiliations:** 1 Department of Pediatrics, Neonatal Division, School of Medicine, University of Jordan, Amman, Jordan; 2 Department of Internal Medicine, School of Medicine, University of Jordan, Amman, Jordan; 3 Department of Public Health, Faculty of Medicine, Jordan University of Science and Technology, Irbid, Jordan; 4 Eastern Mediterranean Public Health Network, Amman, Jordan; 5 Department of Family and Community Medicine, School of Medicine, University of Jordan, Amman, Jordan; 6 Department of Medicine, Infectious Disease Division, School of Medicine, University of Jordan, Amman, Jordan; 7 Department of Computer Science, Al Quds University, Jerusalem, Palestine; 8 Department of Computer Engineering, Istinye University, Istanbul, Türkiye; 9 Laboratory of Molecular Microbiology, Vaccinology and Biotechnology Development (LR16IPT01), Institute Pasteur de Tunis, University of Tunis El Manar, Tunis, Tunisia; 10 Global South Artificial Intelligence for Pandemic and Epidemic Preparedness and Response Network (AI4PEP), Toronto, ON, Canada; 11 Molecular Testing Laboratory, Medical Laboratory Department, Faculty of Health Sciences, Beirut Arab University, Beirut, Lebanon; 12 Department of Nutrition and Food Technology, Faculty of Agriculture, University of Jordan, Amman, Jordan; 13 Department of Human Nutrition, College of Health Sciences, QU-Health, Qatar University, Doha, Qatar; 14 Dahdaleh Institute for Global Health Research, York University, Toronto, ON, Canada; 15 Global South Artificial Intelligence for Pandemic and Epidemic Preparedness and Response Network (AI4PEP), Toronto, ON, Canada; 16 Department of Mathematics and Statistics, Faculty of Science, York University, Toronto, ON, Canada

**Keywords:** Jordan, Palestine, infectious diseases, systematic review, brucellosis, meningitis, respiratory syncytial virus, tuberculosis

## Abstract

**Background:**

Jordan and Palestine face public health challenges due to infectious diseases, with the added detrimental factors of long-term conflict, forced relocation, and lack of resources. Added to these are the increased rates of morbidity and mortality from having limited healthcare services available due to a lack of funding, poor disease surveillance systems, and entrenched systemic weaknesses. The purpose of this systematic review was to report the prevalence of infectious diseases in Jordan and Palestine in order to inform the development of targeted public health programs that use both standard and novel approaches to reduce the region's disease burden.

**Method:**

As defined by the Preferred Reporting Items for Systematic Reviews and Meta-Analyses (PRISMA) guidelines, the review included prospective, retrospective, cross-sectional, and case series studies published from January 2021 to February 2024. Non-English studies were excluded due to resource limitations, as were studies published before the COVID-19 pandemic (i.e., before January 2021) to focus on post-COVID-19-pandemic trends. We used diagnostic techniques (screening, laboratory, and confirmatory tests) to test for microorganisms in adults and children from at-risk populations in Jordan and Palestine. Test-negative controls were contrasted with patients who had positive test results. A manual reference screening process was added to a systematic search of PubMed and Scopus. Full-text, English-language publications published after January 2021 were eligible; protocols, reviews, case reports, and articles written in languages other than English were not. The Rayyan platform was used by two reviewers to independently screen studies. Infection type, causative microorganism, symptoms, mortality, risk factors, seasonal fluctuations, and study details (author, year, location, design, and participant characteristics) were among the extracted data. The Hoy 2012 Checklist was used to evaluate the risk of bias. Open Meta (Analyst) was used to analyze the 13 studies that satisfied the inclusion criteria. Study design, risk of bias, heterogeneity, publication bias, indirection, imprecision, effect size, and residual confounding were all considered when grading the quality of the evidence using the Grading of Recommendations, Assessment, Development, and Evaluation (GRADE) approach.

**Results:**

The results revealed that four studies addressed the prevalence of *Brucella* infection in Jordan and Palestine. The pooled estimate was 42.2% (95% CI: 18.8%–65.6%, *I*^2^: 99.7%, *P:* 0.001, *n* = 4 studies, 4,483 patients). In the studies that addressed diarrhea, in 31 of 159 (19.5%) cases, 20 were caused by *Entamoeba* (12.6%), 10 were caused by *Blastocystis* (6.3%), and 1 (0.6%) was caused by *Cryptosporidium*. As some cases had more than one parasite, the certainty of evidence (COE) was assessed as very low. The pooled estimate for viral causative agents of respiratory tract infections was 60% (95% CI: 11.8%–100%), while for bacterial causes, the pooled estimate was 24.4% (95% CI: 0%–68.3%). Respiratory syncytial virus (RSV) was the most common agent, with a pooled estimate of 57.9% (95% CI: 29.8%–85.9%), while influenza had a pooled estimate of 28.4% (95% CI: 5.3%–51.5%). Furthermore, two studies addressed the prevalence of meningitis in pediatric patients. In adult patients, of 192 patients known to have meningitis, a causative agent was identified in only 86 cases, with 83 (49%) classified as aseptic meningitis.

**Conclusion:**

The review addressed the limitations of diagnostic capacity, reporting systems, and population-level data concerning high-burden and emerging pathogens within Jordan and Palestine. Specifically, the growth in the number of cases with respiratory tract infections, protozoal diarrheal diseases, and brucellosis indicates that improvements in surveillance systems and diagnostic processes need to be standardized and implemented throughout Jordan and Palestine to provide accurate and reliable diagnoses. In addition, improving the quality and quantity of the data and incorporating new technologies and other innovative approaches as a complement to existing public health indicators within Jordan and Palestine would be beneficial for better detecting these diseases at the earliest possible time and would provide the opportunity to establish evidence-based disease management strategies within the region.

## 1 Introduction

Both Jordan and the West Bank of Palestine continue to face significant public health challenges caused by infectious diseases. This is particularly apparent in areas affected by conflict, with limited resources available as a result of displacement and limited access to healthcare. All of these factors result in higher-than-normal incidence and mortality rates for infectious diseases (1, 2). High-income countries have had success in reducing the disease burden through vaccination, clean water, and health systems. These interventions have not had the same impact in lower- and middle-income countries (3, 4). Humanitarian crises often increase vulnerability due to overcrowding and lack of continuity of care from health systems, leading to increased chances of disease spread (5, 6).

Among the many things that affect Jordan and Palestine, unstable political systems, healthcare system inadequacies, and financial constraints are greatly impacted by the current situation. Jordan has one of the largest refugee populations in the world and their refugee camps (Azraq and Zaatari, for example) have poor sanitation and very little access to basic health care services—this puts numerous people at increased risk of contracting waterborne or respiratory diseases (7, 8). Moreover, assaults and mobility restrictions are causing further complications within the Palestinian healthcare system and are resulting in an inability to sufficiently monitor and manage outbreaks (9, 10).

The rise of zoonotic diseases such as brucellosis underscores the importance of environmental, political, and economic factors in the transmission of disease (11, 12).

Most current systems rely on passive case detection and communication via paper reports, and, as noted during the COVID-19 pandemic, these delays are both insufficient and non-comprehensive in terms of available data (13–16). For the purpose of identifying reporting gaps and guiding targeted public health efforts, this assessment examined the prevalence of infectious diseases from January 2021 to February 2024 (9). Disease surveillance, reporting, and epidemic response may be impacted by the differences between Jordan’s and Palestine's public health infrastructures and service delivery, as these are a significant source of bias in determining the incidence of infectious diseases.

## 2 Methods

### 2.1 Design standards and reporting guidelines

This systematic review and meta-analysis aimed to investigate the prevalence of infectious diseases in Jordan and Palestine and adhered to the Preferred Reporting Items for Systematic Reviews and Meta-Analyses (PRISMA) guidelines (17).

### 2.2 Operational definitions

Respiratory tract infection (RTI): an infectious disease affecting the upper or lower respiratory tract that can be proven by clinical or laboratory tests, such as infections caused by bacteria (*Streptococcus pneumoniae* and *Bordetella pertussis*) or viruses [Respiratory syncytial virus (RSV) and influenza], is referred to as a respiratory tract infection.

High-risk population: individuals with increased susceptibility or exposure because of their age (younger than 5 years or older than 65), occupation (such as those who work in healthcare or raise livestock), comorbidities, status as refugees, or restricted access to healthcare.

Causative agent: a particular pathogen, such as a virus, bacterium, or protozoan, that causes an infection.

High-burden diseases, such as brucellosis, tuberculosis (TB), respiratory infections, and waterborne infections, are infectious diseases with significant prevalence, morbidity, or death rates in Jordan and Palestine.

Types of studies

1. The review included prospective cohorts, retrospective cohorts, cross-sectional studies, and case series studies published from January 2021 to February 2024.
2. Studies conducted prior to January 2021 were excluded to focus on post-COVID-19-pandemic trends, while non-English studies were excluded due to resource limitations.

### 2.3 Criteria for inclusion and exclusion

The inclusion and exclusion criteria are presented in Table 1. The inclusion criteria, presented in the Participants, Interventions, Comparator, and Outcome (PICO) format, were as follows:

**Table 1:** Selection criteria for primary research articles on infectious disease prevalence in Jordan and Palestine from January 2021 to February 2024.

Inclusion criteria	Exclusion criteria
Studies conducted in Jordan or Palestine	Non-human studies
Studies reporting the prevalence of at least one infectious disease	Studies conducted outside Jordan or Palestine
Studies involving adult or pediatric populations	Studies without prevalence data COVID-19 studies
Published between January 2021 and February 2024	Studies using non-primary data (systematic reviews, meta-analyses, editorials, protocols, and case reports)
Primary research articles (cross-sectional, cohort, prospective cohorts, retrospective cohorts, studies, case series, and surveillance-based)	Duplicate publications or datasets
	Non-English language studies

*Participants:* Adult and pediatric patients tested for a microorganism/s in a (at-risk) population residing in Jordan or Palestine.

*Intervention:* Diagnostic methods used (specific diagnostic tests, initial screening, and laboratory tests)

*Comparator:* Patients with negative test results (healthy subjects), serving as a control group for those with positive test results (patients).

*Outcome:* Prevalence of infectious diseases in Jordan and Palestine.

The search strategy was a systematic electronic search using PubMed and Scopus. A manual review of references cited in relevant articles was also carried out to identify additional studies. The Boolean search used the following keywords and synonyms: (“Influenza” OR “ Measles” OR “Mumps” OR “Poliomyelitis” OR “Rubella” OR “herpesvirus” OR “Chickenpox” OR “Whooping Cough” OR “Haemophilus influenzae” OR “Respiratory Syncytial Virus” OR “Hepatitis” OR “Diphtheria” OR “Tetanus” OR “Arboviruses” OR “Meningitis” OR “Pertussis” OR “Anthrax” OR “Gonorrhoeae” OR “Syphilis” OR “Scabies” OR “Leprosy” OR “Rabies” OR “Leishmania” OR “Echinococcosis” OR “Schistosomiasis” OR “Brucellosis” OR “Tuberculosis” OR “Human Immunodeficiency Virus” OR “Dengue Fever” OR “Malaria” OR “Oxyuriasis” OR “Viral Meningitis” OR “Cytomegalovirus” OR “Epstein–Barr virus” OR “Trophozoite” OR “Ascariasis” OR (Diarrhea AND (“*Vibrio cholerae*” OR “*Escherichia coli*” OR “*Bacillus cereus*” OR “*Staphylococcus aureus*” OR “*Giardia lamblia*” OR “*Cryptosporidium*” OR “*Cyclospora*” OR “*Shigella*” OR “*Salmonella*” OR “*Campylobacter*” OR “*Clostridium difficile*” OR “*Entamoeba histolytica*”))) AND (“Jordan” OR “Palestine” OR “West Bank” OR “Gaza Strip”) AND (“Spread” OR “Trend” OR “Prevalence” OR “Incidence” OR “Severity” OR “Mortality” OR “Risk Factors” OR “Diagnosis” OR “Symptoms”).

### 2.4 Study screening and selection procedures

Two reviewers used the Rayyan AI-Powered Systematic Review Platform to independently assess the titles and abstracts (18). The reviewers were able to make consistent decisions because they used a predetermined technique. The full texts of all potentially eligible studies were acquired. Recent AI-based frameworks for real-time surveillance of infectious diseases in urban settings have revealed the potential of combining surveillance data with advanced analytics for early epidemic identification (19).

### 2.5 Handling of disagreements

During the title/abstract screening, early disagreements were resolved via dialog. The first author made the final decisions on inclusion if the disagreement continued.

### 2.6 Protocol and guidelines followed

The PRISMA reporting guidelines and a study-specific methodology intended to promote clarity and accessibility throughout the screening procedure were the foundations of this review. Study characteristics, population data, and eligibility requirements were recorded using a standard screening form. Two reviewers separately reviewed each article's title and abstract before assessing the full text of those that met the inclusion requirements. Each article was examined to ensure it satisfied the requirements for inclusion.

The full texts of the publications that met the inclusion requirements were reviewed by two independent reviewers, who confirmed that they were consistent with the eligibility requirements. The inclusion of the remaining cases was decided by the first author, and disagreements amongst reviewers were resolved through discussion.

### 2.7 Data extraction process

We used a structured form for data extraction. Data were extracted and the form was filled out separately by two reviewers, and a third (the first author) verified its completeness and consistency.

### 2.8 Extracted variables

The first author's last name, the year of publication, and the study's location, method, and participant data (such as age demographics, disease type, and treatment) were among the data collected. Additional data collected included the type of infection, causative microorganism, presenting symptoms, and mortality rates. The primary outcome included the prevalence of infectious diseases. Information on risk factors for infectious diseases and seasonal differences was also extracted. Simultaneously, the risk of bias was identified, and data were collected from the included studies. This study included both the general and refugee populations in Jordan and Palestine. When evaluating the surveillance data and assessing infection prevalence, differences between Jordan’s and Palestine's public health systems were considered (20, 21). Information was gathered from public reports, pertinent literature, and national surveillance systems.

### 2.8 Risk of bias assessment

The Hoy 2012 Checklist was utilized to determine the degree of bias. This validated tool for evaluating the quality of prevalence studies was used to evaluate the included studies through 10 questions, with answers including “yes,” “no,” or “not available.” This checklist determined the risk of bias in each study as either low, high, or unclear (22) (see Table A1, in Appendix A).

### 2.9 Data synthesis

For this meta-analysis, we included 13 studies and performed statistical analyses using Open Meta [Analyst] software. The main outcome assessed was the proportion of patients with confirmed infections among those suspected of having an infection. Recognizing the methodological and clinical diversity (due to the observed variability in study designs and populations) inherent across the selected studies, a random-effects model was employed. This method provided a more comprehensive understanding of the data and supported the analysis of differences.

The random-effects model was used for analyses involving more than three trials. A fixed-effects model was used for analyses with three or fewer studies for more accurate results. We computed the prevalence as our main effect and included 95% confidence intervals (CIs) to indicate the accuracy of our estimations. To visualize the results, forest plots were created to show the prevalence estimates and the variability for each outcome. The chi-square test was used to assess the level of consistency across the included studies, with a *p*-value of 0.1 or less indicating a statistically significant level of heterogeneity. Heterogeneity was quantified using an *I*^2^ value, with 0%–40% indicating very low heterogeneity, 30%–60% indicating moderate heterogeneity, 50%–90% indicating high heterogeneity, and 75%–100% indicating very high heterogeneity.

### 2.10 Justification for the meta-analysis

Despite being primarily focused on the systematic review, this study also performed a meta-analysis to determine the total prevalence of infectious diseases. Although there were concerns over heterogeneity among the studies, such as differences in the methods used for diagnosis, variability in population characteristics, and differences in research design, it was deemed appropriate to use the information gathered from this meta-analysis to present an overall estimate of the prevalence of certain infectious diseases in a given geographical area. We should mention that there was high heterogeneity in the studies we included, which is a common problem when integrating dissimilar data from studies with dissimilar approaches. However, by using a random-effects model, we accounted for differences among the studies, as this model is suitable for circumstances where study characteristics differ. This approach allowed us to combine data from studies with different designs, diagnostic techniques, and population groups and calculate the overall trend in infectious disease prevalence in Jordan and Palestine.

### 2.11 Assessment of the quality of evidence

#### 2.11.1 Quality of evidence

We used the Grading of Recommendations, Assessment, Development, and Evaluation (GRADE) approach to evaluate the quality of the evidence (23). This method considered the study structure, risk of bias, inconsistency (heterogeneity), indirectness (answer directness), imprecision (small sample size, wide CI), publication bias, presence of magnitude of effects, and presence of residual confounding for the assessment of the quality of evidence for each outcome, with four levels of quality: high (⊕⊕ ⊕⊕), moderate (⊕⊕⊕◯), low (⊕⊕ ◯◯), and very low (⊕◯◯◯).

This systematic review was not registered in Prospero because it focuses on prevalence estimates and epidemiological evaluation, which do not align with Prospero's registration criteria for evaluating specific health interventions.

## 3 Results

**Figure 1:**
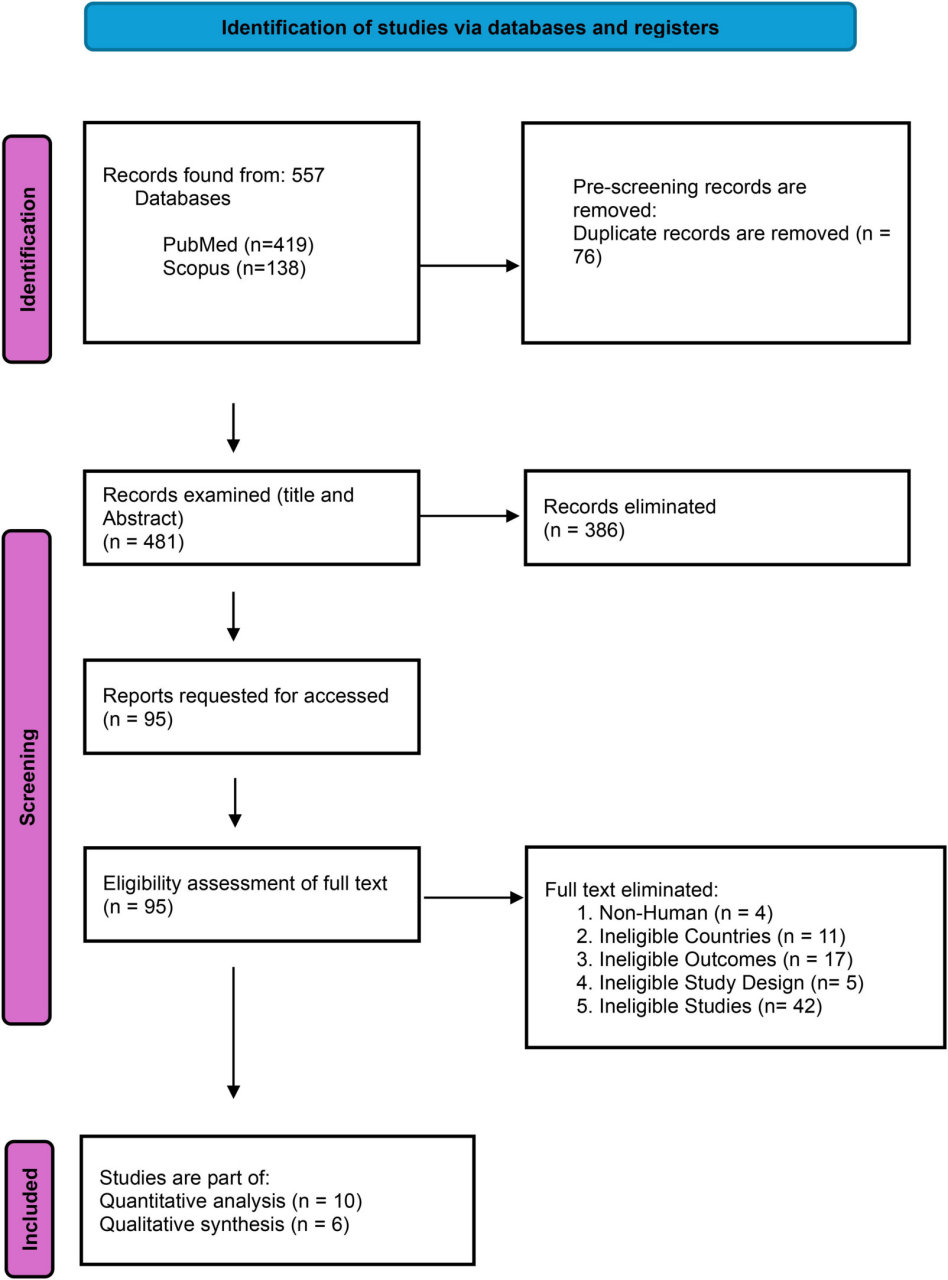
PRISMA flow diagram for the study inclusion process.

A total of 557 records—419 from PubMed and 138 from Scopus—were gathered and screened for eligibility, with 481 studies remaining after duplicates were removed. We further excluded 386 records after screening their abstracts and titles. After reviewing 95 full-text publications, 16 were chosen for qualitative and quantitative synthesis. The details of the included studies are presented in Figure 1.

The 16 included studies (7, 24–38) are summarized in Table 2, presenting information on the study's author, year, country, design, and duration; the age, population, and size of the sample; the microorganisms assessed; and the diagnostic techniques used. The review comprises 11 retrospective cohort studies, three cross-sectional studies, and two prospective cohort studies. The study periods span from 2021 to 2024. For most outcomes, the quality of the evidence was rated as extremely low because of uncertainty related to bias, imprecision, and inconsistency.

**Table 2:** Characteristics of the included studies that investigated infectious disease prevalence in Jordan and Palestine.

Study (author, publication Year)	Country	Study design	Study period	Age of participants (mean/range)	Population type	Sample Size (*n*)	Target microorganisms	Diagnostic methods
Al-Amr et al. (21)	Jordan	Retrospective cohort	2016–2020	Mean age: 35.5 years	General population	1,497	*Brucella*	Rapid slide agglutination test (IgG, IgM).
Al-Sanouri et al. (22)	Jordan	Cross-sectional	2022	Median Age: 30 years	Syrian refugees	1,562	*Brucella*	ELISA IgG *Enzyme-Linked immunosorbents Assay.*
Aljanazreh et al. (23)	Palestine	Retrospective cohort	2015–2017	Mean age: 25.2 ± 17.5	General population	1,324	*Brucella*	Agglutination Rose Bengal Test (RBT)
Alzuheir et al. (24)	Palestine	Cross-sectional	2020	Range: 24–55 years	Veterinarians	100	*Brucella*	ELISA IgG
Hijjawiet al. (25)	Jordan	Cross-sectional	2014–2016	Range: 1 month to 54 years	General population	159	*Entamoeba, Blastocystis, and Cryptosporidium*	Polymerase chain reaction (PCR)
Abu Seir et al. (27)	Palestine	Retrospective cohort	2011–2016	32.8 years	Hospitalized patients with respiratory tract infections	15,413	RTI causative agents	Not reported
Oweidat et al. (27)	Jordan	Retrospective cohort	2017–2020	NA	Hospitalized patients	695	*RTI causative agents*	PCR on nasal/throat swab
Khasawneh et al. (28)	Jordan	Retrospective cohort	2021–2022	32.77	Patients with respiratory tract infection symptoms	339	*RTI causative agents*	Combined oropharyngeal/n nasopharyngeal swabs
Masri et al. (29)	Jordan	Retrospective cohort	2016–2020	Range: 1 month to 14 years	Patients with aseptic meningitis	131	*Enterovirus, Varicella zoster,* and mumps	Viral CSF PCR
Mohialdin et al. (30)	Jordan	Retrospective cohort	2019–2022	NA	Pediatric patients admitted with suspected meningitis	332	Multiple	Not reported
Khader et al. (31)	Jordan	Retrospective cohort	2016–2020	30.1 (17.2) years	General population	1,711	Tuberculosis	Clinically diagnosed tuberculosis, i.e., Positive on smear microscopy, culture, or WHO-approved rapid test, such as GeneXpert MTB/RIF assays.
Al-Zayadneh et al. (32)	Jordan	Retrospective cohort	2018–2019	2.1 Years	Hospitalized patients with respiratory tract infections	152	RTI causative agents	PCR on nasal/throat swab
Freeman et al. (33)	Jordan	Prospective cohort	2010–2012	Median: 3.2 Years	Hospitalized patients with fever and/or respiratory symptoms	2,365	Respiratory syncytial virus (RSV)	RSV rapid diagnostic assays or PCR RSV
Halasa et al. (34)	Jordan	Prospective cohort	2010–2013	Median: 0.291667 years	Children <2 years old with respiratory symptoms and/or fever	3,168	Multiple	Nasal/throat swabs, RT/PCR on nasal/throat swabs
Abuhayyeh et al. (35)	Jordan	Retrospective cohort	2015–2019	Range: 1–27.3 years	Patients diagnosed with meningitis	192	Multiple	CSF analysis, culture and gram staining, blood culture, and CSF PCR
Amro et al. (36)	Palestine	Retrospective cohort	2000–2020	25.9–16.9	General population	7,935	*Brucella*	Not reported

ELISA, enzyme-linked immunosorbent assay.

The microorganisms assessed included *Entamoeba*, *Blastocystis*, *Cryptosporidium*, *Brucella*, RTI causative agents, *Enterovirus*, *Varicella zoster*, mumps, tuberculosis, and RSV. The studies were categorized according to the following five clinical presentations: febrile disease, diarrhea, upper respiratory tract infections, meningitis, and tuberculosis.

Studies addressing febrile disease:

1. Studies that addressed *Brucella* infection

**Figure 2:**
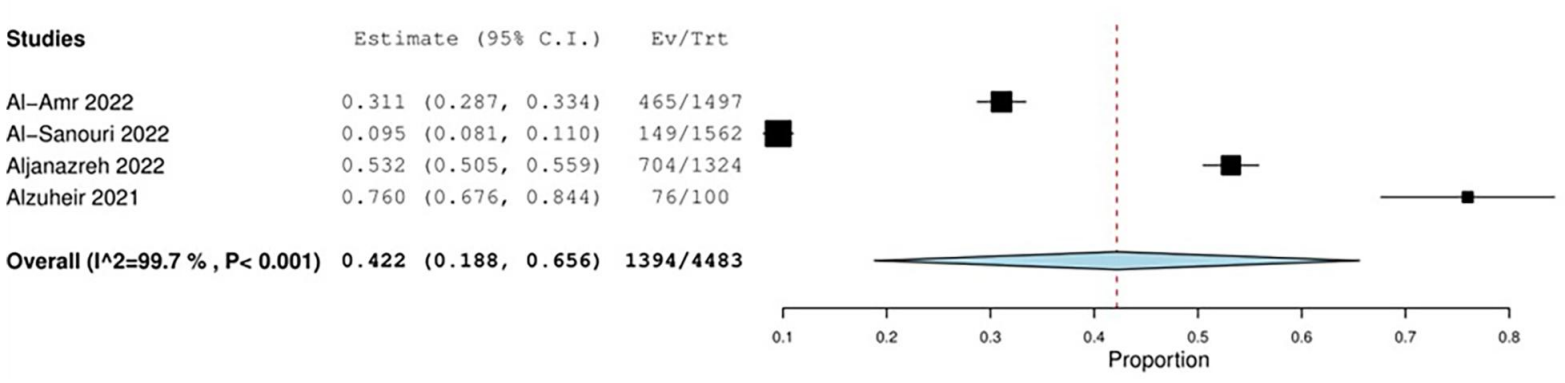
The prevalence of *Brucella* infection in Jordan and Palestine.

Four studies addressed the prevalence of *Brucella* infection in Jordan and Palestine. The pooled prevalence estimate was 42.2% (95% CI: 18.8%–65.6%, *I*^2^: 99.7%, *P* 0.001, *n* = 4 studies, 4,483 patients), as shown in Figure 2.

Two of the included studies, Al-Amr et al. and Al-Sanouri et al. (24, 25), were conducted in Jordan. The other two, Al-Zuheir et al. and Aljanazreh et al. (26, 27), were conducted in Palestine. Within these studies, differences in prevalence were observed according to the population’s type, age, geographical location, and exposure to livestock.

There were differences in the populations in the studies, as Al-Amr et al. and Aljanazreh et al. (24, 26) focused on the general populations of Jordan and Palestine, respectively, while Al-Zuheir et al. (21) focused on veterinarians who work with livestock and Al-Sanouri et al. studied brucellosis in Syrian refugees residing in Jordan. Amro et al. (7) reported that the incidence of brucellosis in the West Bank from 2000 to 2020 was 9.4 cases per 100,000. The COE was assessed as very low (⊕ ◯◯◯) due to risk of bias concerns, imprecision, and inconsistency.

2. Studies that addressed diarrhea

The study by Hijjawi et al. (28) investigated the prevalence of diarrhea caused by parasites in Jordan. The three microorganisms of interest were *Entamoeba*, *Blastocystis*, and *Cryptosporidium*. The overall prevalence of diarrhea due to these parasites was 31 out of 159 (19.5%), with 20 caused by *Entamoeba* (12.6%), 10 caused by *Blastocystis* (6.3%), and 1 (0.6%) caused by *Cryptosporidium*. Some had more than one parasite. The COE was assessed as very low (⊕◯◯◯) due to risk of bias concerns, imprecision, and inconsistency. There were no combined infections with the three tested parasites in the same patient in this study.

3. Studies that addressed respiratory tract infections

Seven studies evaluated viral and bacterial pathogens that cause RTIs among pediatric and adult populations, including 22,132 patients. The key findings were as follows.

### 3.1 Respiratory tract infections in adults

**Figure 3:**
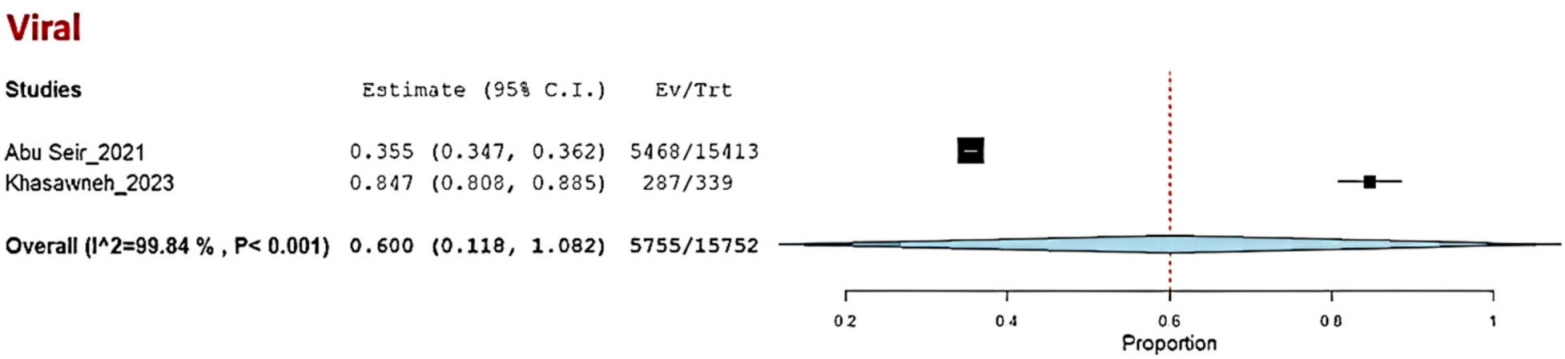
The pooled prevalence estimate for respiratory infection due to viral causes in adults.

**Figure 4:**
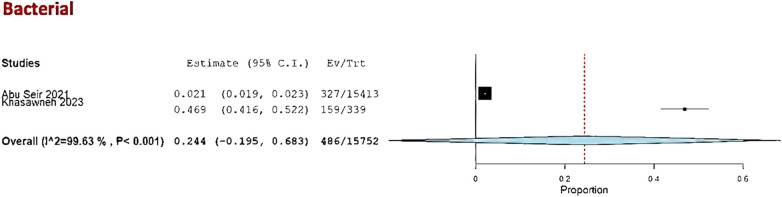
The pooled prevalence estimate for respiratory infection due to bacterial causes in adults.

Three studies—Abu Seir et al. (29), Oweidat et al. (30), and Khasawneh et al. (31)—examined the prevalence of RTI causative agents in Jordan and Palestine among adults, with a total of 16,447 adult patients included. All three studies focused on patients presenting to tertiary hospitals with suspected RTIs. In these studies, influenza, *Enterovirus*, RSV, *Bordetella*, adenoviruses, and *S. pneumoniae* were examined as causative agents. The COE was assessed as very low (⊕◯◯◯) due to concerns about risk of bias and inconsistency. Among these studies, the pooled estimate for viral causative agents was 60% (95% CI: 11.8%–100%) (29, 31) (Figure 3), while the pooled estimate was 24.4% (95% CI: 0%–68.3%) for bacterial causes (29, 31) (Figure 4).

**Figure 5:**
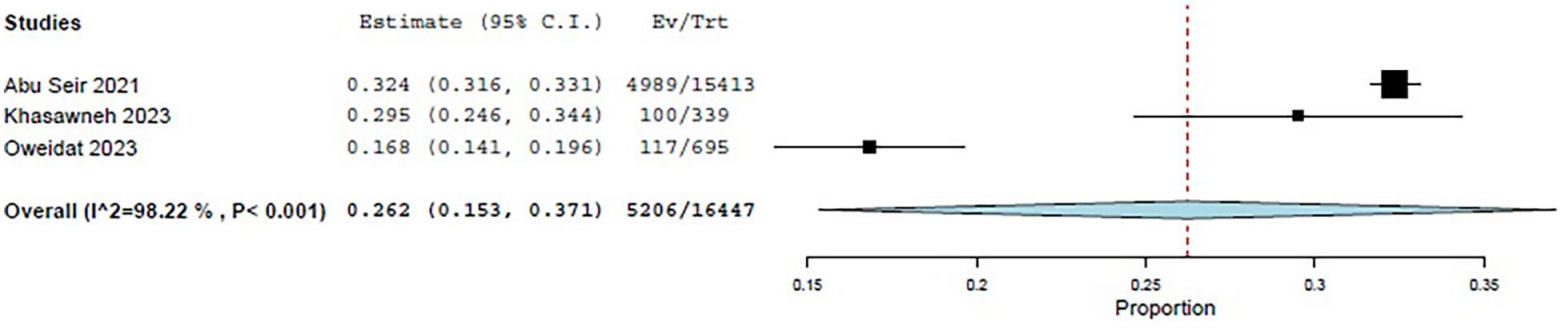
The pooled prevalence estimate for influenza viral agents.

Among the viral agents in adults, influenza had the highest prevalence, with a pooled estimate of 26.2% (95% CI: 15.3%–37.1%) (29–31) (Figure 5).

**Figure 6:**
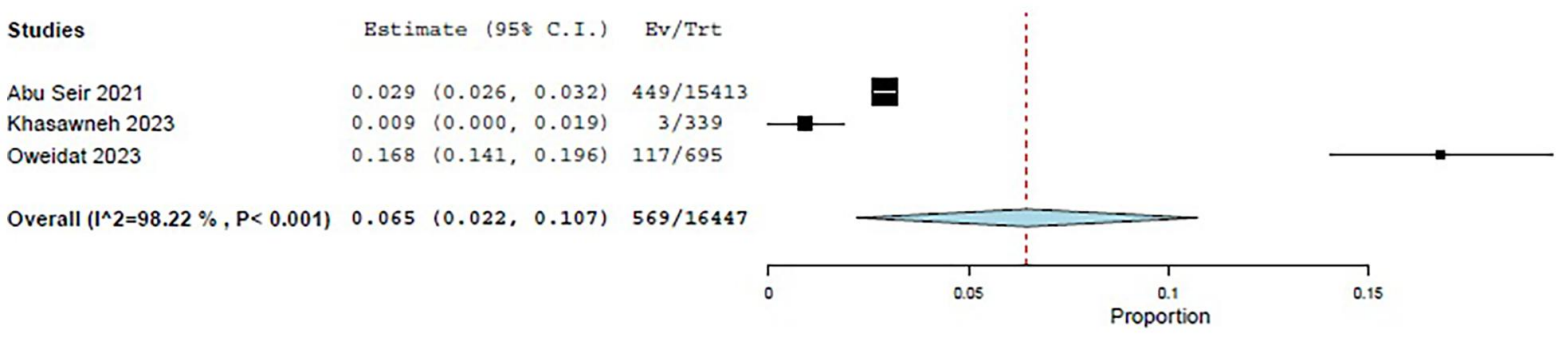
The pooled prevalence estimate for viral RSV.

RSV had a pooled estimate of 6.5% (95% CI: 2.2%–10.7%) (29–31) (Figure 6).

**Figure 7:**
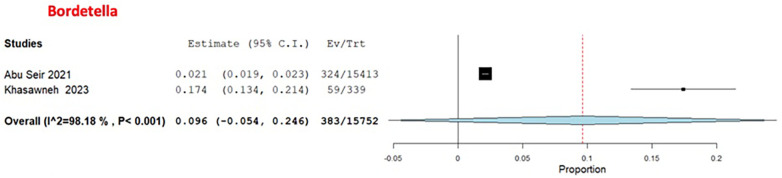
The pooled prevalence estimate for *Bordetella*.

**Figure 8:**
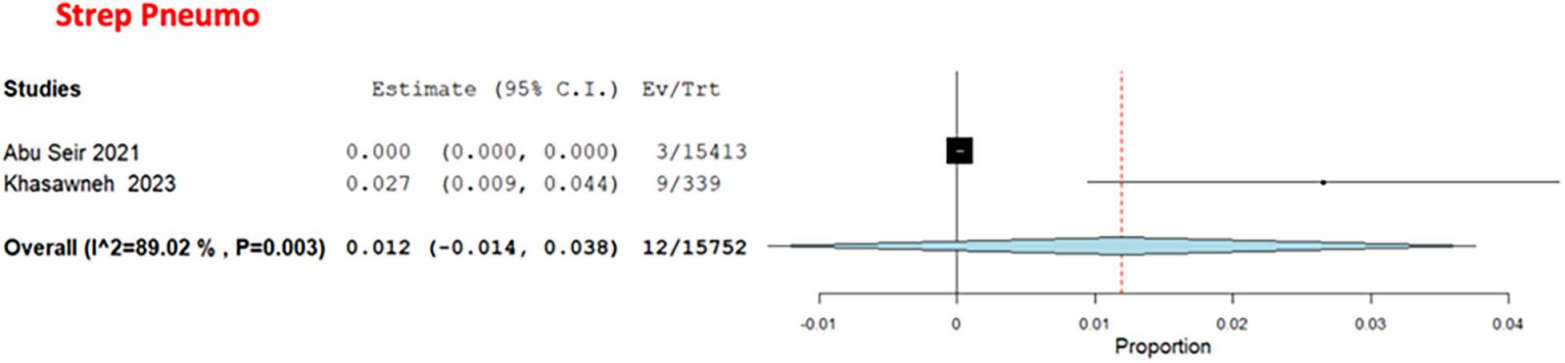
The pooled prevalence estimate for *S. pneumoniae* pneumonia.

Among the bacterial causes in adults, *Bordetella* was the most common, with a pooled estimate of 9.6% (95% CI: 0%–24.6%) (29, 31) (Figure 7). *S. pneumoniae* had a pooled estimate of 1.2% (95% CI: 0%–3.8%) (29, 31) (Figure 8).

**Figure 9:**
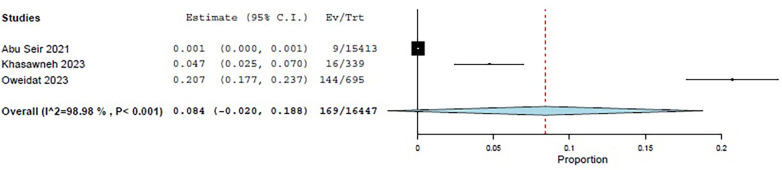
The pooled prevalence estimate for *Enterovirus* among adults.

**Figure 10:**
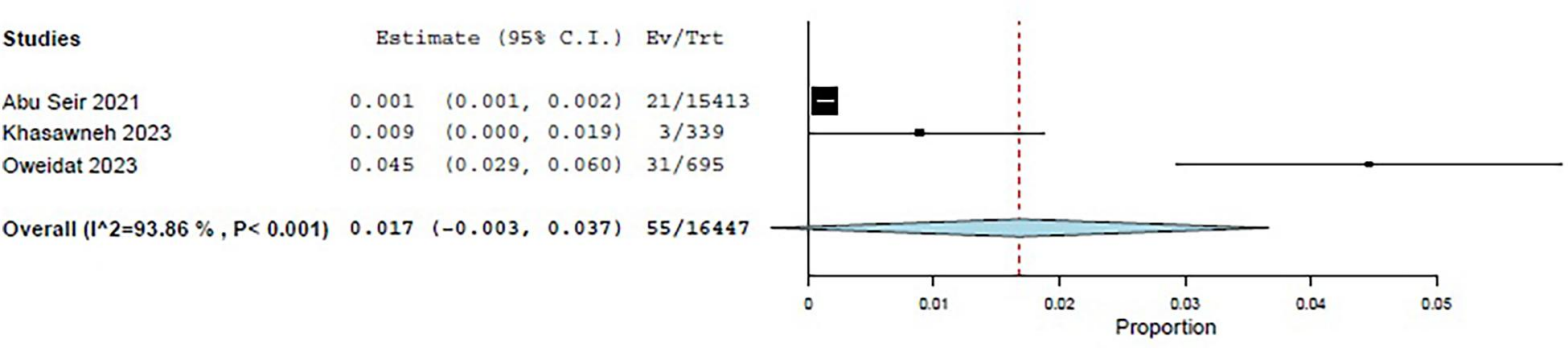
The pooled prevalence estimate for adenovirus among adults.

For adults, the pooled estimate for *Enterovirus* was 8.4% (95% CI: 0%–18.8%) (29–31) (Figure 9), and for adenovirus, it was 1.7% (95% CI: 0%–3.7%) (29–31) (Figure 10).

### 3.2 RTI causative agents in the pediatric population

**Figure 11:**
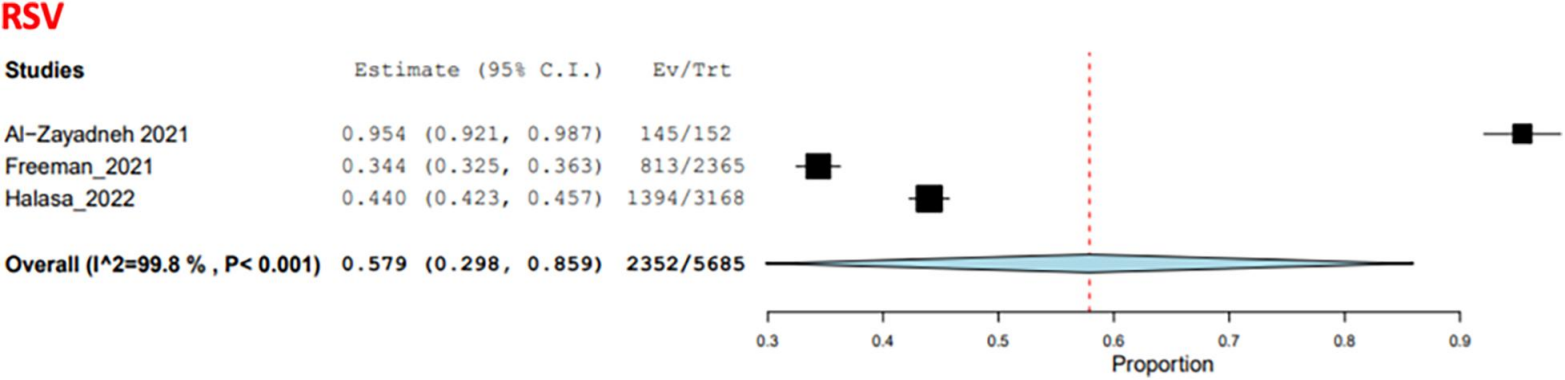
The pooled prevalence estimate for respiratory infection due to RSV in the pediatric population.

**Figure 12:**
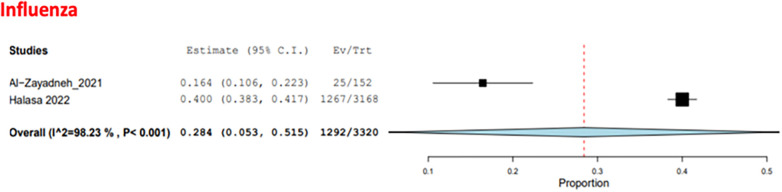
The pooled prevalence estimate for respiratory infection due to influenza in the pediatric population.

Three studies examined the frequency of RTI-causing agents in the pediatric population (35–37), with a total of 5,685 patients. The two causative agents addressed in these studies were RSV and influenza. The COE was assessed as very low (⊕ ◯◯◯) due to risk of bias concerns and inconsistency. RSV was the most common agent, with a pooled estimate of 57.9% (95% CI: 29.8%–85.9%) (Figure 11), while influenza had a pooled estimate of 28.4% (95% CI: 5.3%–51.5%) (35, 37) (Figure 12).

4 Studies that addressed meningitis

One study addressed the prevalence of meningitis in adult patients, while two studies addressed the prevalence of meningitis in pediatric patients. For adult patients, Abuhayyeh et al. (38) investigated the causative agent in 192 patients known to have meningitis. The causative agent of the meningitis was identified in only 86 cases, with the remaining 83 (49%) cases labeled as aseptic meningitis. These causative agents included *Enterovirus* (most common), herpes simplex virus (HSV) 1 or 2, Epstein–Barr virus (EBV)*,* cytomegalovirus (*CMV), S. pneumoniae*, *Mycobacterium tuberculosis*, and *Klebsiella pneumoniae*.

The COE was assessed as very low (⊕ ◯◯◯) due to risk of bias concerns and inconsistency. There is a strong need to improve diagnostic accuracy to ensure timely and appropriate identification of cases.

Masri et al. (32) examined how many pediatric patients diagnosed with aseptic Meningitis in Jordan also tested positive for *Enterovirus*, varicella-zoster virus, or mumps virus. The total prevalence of these four viruses was 50% (66 of 131 cases). *Enterovirus* was the leading agent and was identified in 60/131 (45.8%) cases. In a study carried out by Mohialdin et al. (33), it was determined that most of the subjects within the study population (265 of 332 or 79.8%) had a diagnosis of a non-specific kind of meningitis. Bacterial meningitis was definitively diagnosed among 12.3% of those involved in the study (*n* = 41), while there were viral (aseptic) cases present in 7.8% of individuals (*n* = 26). There was concern regarding bias and inconsistencies, so the COE is rated as very low (⊕ ◯◯◯).

5 Studies that addressed tuberculosis

Khader et al. (34) examined the occurrence of tuberculosis within Jordan and showed that from 2016 to 2020, the average annual incidence rate was 3.32 cases per 100,000 population. Due to differences in study design and possible sources of bias, the COE was rated as very low (⊕ ◯◯◯).

#### 3.2.1 Heterogeneity observations

The reviewed studies had a significant amount of heterogeneity (*I*² > 99%), largely due to differences in the locations, demographic characteristics of the populations, and diagnostic methods used across studies. This variability was accounted for using a random-effects model, which allowed for adequate balancing of study variability. Although the variability of the studies presents research challenges, it is also a reflection of the differences in epidemiological patterns in that area, which indicates the need for the standardization of future studies.

## 4 Discussion

The research showed that both Jordan and Palestine had identified significant weaknesses in the communication of surveillance concerning infectious diseases, showing the need for improvement in public health systems. Brucellosis is still an endemic disease and has shown to be especially problematic in human populations due to commercial livestock trading being poorly controlled and occupational exposure to *Brucella*-infected animals (7, 8, 39). Waterborne diseases continue to be very prevalent in both Jordan and Palestine due to a lack of sufficient hygiene standards and limited access to potable water (28). Respiratory infections, including influenza and RSV, are the most prevalent seasonal Diseases. Although artificial intelligence (AI) and machine learning could increase both the detection of outbreaks and the allocation of resources, the infrastructure required for the effective surveillance of outbreaks, standardized diagnostic procedures, and increased public health capacity must be significantly improved (40, 41).

### 4.1 Febrile diseases and brucellosis

In febrile patients, the pooled prevalence of brucellosis in Jordan amounted to 42.2%, considerably higher than the prevalence rates reported in Uganda (14.9%) and Tanzania (6.1%) (42, 43). The relatively high prevalence of brucellosis in Jordan can be attributed to extensive exposure practices, including the consumption of unpasteurized dairy products, high levels of livestock husbandry, and unsafe animal transportation. The majority of those impacted are between the ages of 15 and 29, and the findings align with regional patterns of occupational exposure (24, 39). AI-assisted methods, such as system dynamics modeling, have shown promise in predicting brucellosis outbreaks and evaluating treatments in Jordan (41). The overall number of brucellosis cases in the world is thought to be much higher than what is reported each year. Furthermore, there is a great deal of variation in reporting, surveillance, and diagnosis systems around the world (44).

A probable decrease of 37% in human cases and 48% in animal cases was found using the One Health Model (45, 46). Geographically, Hebron, Palestine, had the highest burden, accounting for 77% of the regional cases (45.6 per 100,000 annually) (7), and in the Mafraq governorate of Jordan, the seroprevalence rate was close to 33.3% (39). Endemicity is still persistent in Lebanon, Syria, Egypt, and Iraq, as shown in comparative studies (47–50).

### 4.2 Waterborne infections and acute diarrhea

In comparison to Ethiopia, which has a prevalence of 16.6%, and China, which has a 1.67% prevalence, 19% of the population in Jordan is infected with protozoans, making it the country with the highest prevalence of protozoal infections (28, 51, 52). This finding indicates that it is imperative to improve environmental conditions and establish surveillance systems to monitor the sanitation and water quality in Jordan to reduce the number of cases of diarrheal diseases.

### 4.3 Respiratory tract infections

Respiratory infections, such as influenza, RSV, and pneumonia caused by bacteria, remain among the major contributors to the increased disease burden due to their high prevalence in Jordan and Palestine (29–31, 35–37). The data from this study show that the RSV prevalence among the children diagnosed with RTIs was 57.9%, indicating that RSV was a considerable burden on the healthcare system within the region. These findings agree with previous national and regional studies. In Jordan, the incidence rate of RSV during the 2006–2007 season was 64% (67–69). The incidence rates remained high (47%) through subsequent years (50, 51), and similar incidences have been reported in nearby countries, such as Lebanon (19%; 53) and Qatar (19.7%; 31, 53, 54). Therefore, RSV continues to be widespread in the Middle East.

*Bordetella* and *S. pneumoniae* have emerged as the most prevalent bacterial agents that cause respiratory disease in adults, even in the context of the limited research that is focused on bacterial respiratory infections in adults. As a result of this significant absence of data on bacterial respiratory infections, it is critical to perform periodic surveillance of these infections in adults.

The epidemiology of viral respiratory tract infections was significantly affected by the COVID-19 pandemic. According to the increasing prevalence of non-pharmaceutical strategies, such as mask use, reduced outdoor activities, and physical separation, the occurrence of influenza dramatically declined during this time (55). Similar decreases in influenza activity were observed worldwide, particularly in South Korea, Singapore, and Japan (55–57, 58, 70).

### 4.4 Meningitis and tuberculosis

The majority of the meningitis cases in Jordan and Palestine were aseptic meningitis, mainly enteroviral, and prehospital antibiotic use is often associated with misdiagnosis (71–75). The most common bacterial pathogen was *S. pneumoniae*. The West Bank/Gaza Strip (1 per 100,000) and Jordan (3 per 100,000) have relatively low prevalence levels of TB (59, 60, 76). This low prevalence could be explained by disease control, supported by increased Bacillus Calmette–Guérin (BCG) vaccination coverage (>93%) and targeted screening through collaborations between the National Tuberculosis Program and the International Organization for Migration (NTP-IOM) (61, 62, 77, 79), and a low incidence of HIV (63). COVID-19-pandemic-related service issues also decreased tuberculosis reports, probably disguising the true prevalence (80).

### 4.5 Limitations and future directions

Although the significant variability (I² > 99%) limits its accuracy and generalizability, this review provides the first regional synthesis of infectious disease prevalence in Jordan and Palestine. Disparities in demographics, indicators, diagnostic techniques, and study design all had an impact on the variability. Despite the recognized risks, a few studies provided data on refugees, and the majority were hospital-based, which limited the representation of rural and poor regions (76–79). An underestimation of the actual disease burden could have resulted from the limited availability of high-quality surveillance data and the potential publication bias.

Future research must include improved data collection techniques, integrated regional surveillance systems, and standardized diagnostic criteria. An AI-driven surveillance system could improve early diagnosis, resource allocation, and prognosis for conditions such as meningitis, tuberculosis, brucellosis, and respiratory infections (16, 45, 64, 65).

## 5 Conclusion and implications

This article provides an updated evaluation of the prevalence of infectious diseases in Jordan and Palestine, highlighting the persistent gaps in data quality and surveillance capabilities due to these areas being affected by conflict and having limited resources. The precision of these findings was reduced by the high heterogeneity among the studies, as the research depends on hospital-based data, limited reporting, and inadequate coverage of vulnerable populations. There are still large gaps in research on many other infectious diseases that emerge in the public health system and on the overall lack of public health surveillance capabilities. The two most critical infectious diseases currently are brucellosis and tuberculosis.

In order to address these problems, we must standardize diagnostic procedures, increase the quality and quantity of national reporting systems, and develop a digital health infrastructure. Jordan's Economic Modernization Vision prioritizes improved health governance and digital transformation (66), thus meeting these requirements, and has the possibility of improving epidemiological surveillance and early outbreak identification in both countries.

## Data Availability

The original contributions presented in the study are included in the article/Supplementary Material, further inquiries can be directed to the corresponding author.

## Appendix

**Table A1:** Hoy checklist for quality and risk of bias assessment of prevalence studies.

Criteria	Rating: Low risk of bias, high risk of bias, or unclear risk of bias
Was the study's target population a close representation of the national population in relation to relevant variables?	
Was the sampling frame a true or close representation of the target population?	
Was some form of random selection used to select the sample, OR was a census undertaken?	
Was the likelihood of nonresponse bias minimal?	
Were data collected directly from the subjects (as opposed to a proxy)?	
Was an accepted case definition used in the study?	
Was the study instrument that measured the parameter of interest shown to have validity and reliability?	
Was the same mode of data collection used for all subjects?	
Was the length of the shortest prevalence period for the parameter of interest appropriate?	
Were the number(s) and denominator(s) for the parameter of interest appropriate?	
Overall quality rating: low or high risk of bias	
